# Supplementary material for: Content-rich biological network constructed by mining PubMed abstracts
Source: BMC Bioinformatics. 2004 Oct 8;5:147. doi: 10.1186/1471-2105-5-147 (PMC528731; doi:10.1186/1471-2105-5-147)
Supplement: Additional File 2 — The original results of the above study (non-essential files are deleted to keep the file size under the limit set by BMC bioinformatics). [file 1471-2105-5-147-S2.bz2 › chilibotAdditionalFile2/dip10/758ID11500384E18144/html/DNAA_REPA.html]

 


 **DNAA** and **REPA** 
  
Found 61 abstracts in PubMed, retrieved 10.  
 

 What does Google say? 
 PDF only 
| .edu only 

---

**Interactive relationship** (e.g. stimulation, inhibition, etc)

**Neutral relationship**- Thus, the result shows that physical tethering, either non covalent or covalent, of domain I and IV of  **DnaA**  and interaction of both domains with  **RepA** , are critical for replication initiation.  Ref: 11500384 EMBO J, 2001
- Neither domain I nor IV of  **DnaA**  could individually collaborate with  **RepA**  to promote pSC101 replication.  Ref: 11500384 EMBO J, 2001
- The N terminal, but not the C terminal, region of  **RepA**  interacted with  **DnaA**  in vitro.  Ref: 11500384 EMBO J, 2001
- None of these mutations drastically changed the structure or thermal stability of the wild type  **RepA**  protein, but in all cases an enhanced interaction with host encoded  **DnaA**  protein was detected by gel filtration chromatography.  Ref: 12562807 J Bacteriol, 2003
- We propose possible explanations for the host range effect of the different  **repA**  mutants, including the enhancement of limiting interactions of  **RepA**  with specific host replication factors such as  **DnaA** .  Ref: 12562807 J Bacteriol, 2003
- Mechanistic aspects of  **DnaA**   **RepA**  interaction as revealed by yeast forward and reverse two hybrid analysis.  Ref: 11500384 EMBO J, 2001
- Using yeast forward and reverse two hybrid analysis and biochemical techniques, we present novel and definitive in vivo and in vitro evidence that both the N terminal domain I and C terminal domain IV of the host encoded  **DnaA**  initiator protein of Escherichia coli interact physically with plasmid encoded  **RepA**  initiator of pSC101.  Ref: 11500384 EMBO J, 2001
- Binding of  **RepA**  to the  **RepA**  boxes was ordered and sequential, with the box closest to the  **DnaA**  binding site box 1 occupied first and the most distant boxes boxes 3 and 4 occupied last.  Ref: 12644491 J Bacteriol, 2003
- The iteron like region was identified upstream of the  **repA**  gene and consisted of two clusters of repeated sequences 17 bp long separated by a putative  **DnaA**  box.  Ref: 11407917 Plasmid, 2001

**Non-interactive relationship** (e.g. studied together, co-existance, homology, etc.)

- Although, interaction of  **DnaA**  with DnaB could not substitute for  **RepA**  DnaB interaction for helicase loading,  **DnaA**  along with integration host factor, DnaC, and  **RepA**  was essential for helicase loading.  Ref: 9874774 Proc Natl Acad Sci U S A, 1999
- The  **DnaA**  replication initiation protein  [ **REPA** ]  has been shown to be essential for DNA strand opening at the AT rich region of the replication origin of the Escherichia coli chromosome as well as serving to recruit and position the DnaB replicative helicase at this open region.  Ref: 12835421 Proc Natl Acad Sci U S A< MedlineTA>Proc Natl Acad Sci U S A, 1999
- Although many bacterial chromosomes require only one replication initiator protein, e.g.,  **DnaA** , most plasmid replicons depend on dual initiators host encoded  **DnaA**  and plasmid encoded Rep  [ **REPA** ]  initiator protein for replication initiation.  Ref: 9874774 Proc Natl Acad Sci U S A, 1999
- To our knowledge, this work reports the first chromosome mutant described in the literature that allows the host range broadening of a plasmid, highlights the essential role played by  **DnaA**  in the establishment of pPS10 replicon in E. coli and provides support for the hypothesis that interactions between  **RepA**  and  **DnaA**  modulate the establishment of pPS10 in that bacteria and probably in other species.  Ref: 12366845 Mol Microbiol, 2002
- Moreover, neither replicon requires the host  **DnaA** , DnaG, and Rep  [ **REPA** ]  functions.  Ref: 10438819 J Virol, 1999
